# Epidemiological characteristics of pulmonary tuberculosis in mainland China from 2004 to 2015: a model-based analysis

**DOI:** 10.1186/s12889-019-6544-4

**Published:** 2019-02-21

**Authors:** Zuiyuan Guo, Dan Xiao, Xiuhong Wang, Yayu Wang, Tiecheng Yan

**Affiliations:** 1 Department of Disease Control, Center for Disease Control and Prevention in Northern Theater Command of the People’s Liberation Army, Shenyang, China; 2 China National Clinical Research Center for Neurological Diseases, Beijing Tian Tan Hospital, No. 119, South 4th Ring Road West, Fengtai District, Beijing, China

**Keywords:** Pulmonary tuberculosis, Mathematical model, Public health, Seasonal fluctuation

## Abstract

**Background:**

We used data released by the government to analyze the epidemiological distribution of pulmonary tuberculosis in mainland China from 2004 to 2015, in order to provide a deeper understanding of trends in the epidemiology of pulmonary tuberculosis in China and a theoretical basis to assess the effectiveness of government interventions and develop more targeted prevention and control strategies.

**Methods:**

A discrete dynamic model was designed based on the epidemiological characteristics of pulmonary tuberculosis and fitted to data published by the government to estimate changes in indicators such as adequate contact rate, prevalence of non-treated pulmonary tuberculosis (abbreviated as prevalence), and infection rate. Finally, we performed sensitivity analyses of the effects of parameters on the population infection rate.

**Results:**

The epidemiological features of pulmonary tuberculosis in China include a pattern of seasonal fluctuations, with the highest rates of infection in autumn and winter. The adequate contact rate has increased slightly from an average of 0.12/month in 2010 to an average of 0.21/month in 2015. The prevalence in the population has continued to decrease from 3.4% in early 2004 to 1.7% in late 2015. The *Mycobacterium tuberculosis* (*M. tuberculosis*) infection rate in the population decreased gradually from 42.3% at the beginning of 2004 to 36.7% at the end of 2015. The actual number of new infections gradually decreased from 1,300,000/year in 2010 to 1,100,000/year in 2015. The actual number of new patients each year has been relatively stable since 2010 and remains at approximately 2,600,000/year.

**Conclusions:**

The population prevalence and the *M. tuberculosis* infection rate have decreased year by year since 2004, indicating that the tuberculosis epidemic in China has been effectively controlled. However, pulmonary tuberculosis has become increasingly contagious since 2010. China should focus on the prevention and control of pulmonary tuberculosis during autumn and winter.

**Electronic supplementary material:**

The online version of this article (10.1186/s12889-019-6544-4) contains supplementary material, which is available to authorized users.

## Background

China has the second highest burden of pulmonary tuberculosis worldwide [1]. Since 2004, pulmonary tuberculosis has been consistently ranked as having the highest number of newly diagnosed patients among all respiratory infectious diseases listed in the statutory notification system of infectious diseases in China [2]. According to the National Tuberculosis Control Program (2011–2015), with the widespread implementation of China’s modern control strategy including early detection of patients, increasing the treatment level, and increasing public awareness, prevention and control measures have achieved remarkable results, and the epidemic has been effectively controlled. From 1990 to 2010, the prevalence of smear-positive tuberculosis decreased from 170 cases to 59 cases per 100,000 of the population [3]. However, the burden, especially of drug-resistant pulmonary tuberculosis, remained high [4–6]. Thus, pulmonary tuberculosis remains a severe, highly infectious respiratory disease with a high population susceptibility and is difficult to treat [7, 8].

In 1979, 1985, 1990, 2000, and 2010, China conducted five national epidemiological surveys of tuberculosis to assess the epidemiological features, such as infection, morbidity, and mortality [4]. However, the data obtained from the surveys are from cross-sectional surveys with limited time points. Moreover, only the population infection rate, population prevalence, and epidemiological distribution characteristics such as patient gender, age, and region were assessed. There was no assessment of trends or changes over time, and the surveys were unable to estimate the adequate contact rate (the number of persons infected by one contagious individual per month in an entirely susceptible population), the number of newly infected persons and new cases per month, as well as other important indicators of the severity of the epidemic. The lack of these indicators makes it difficult to formulate an adequate and accurate judgment of the epidemic.

Assessing these factors requires the use of mathematical models. Many scholars have used mathematical models to analyze the prevalence of pulmonary tuberculosis in China. For example, Li XX et al. established a time series model to analyze the seasonal variation in the number of patients with active tuberculosis in China from 2005 to 2012 [9]; Mehra M et al. established a dynamic model to predict the number of people infected with multidrug-resistant tuberculosis (MDR-TB, which is resistant to at least isoniazid and rifampicin) [10]; and Hu XL et al. and Liu LJ et al. used dynamic models incorporating the effect of seasonal fluctuations to predict national tuberculosis epidemic trends [11, 12]. These models are essential tools for the quantitative analysis of the distribution of pulmonary tuberculosis in China. However, previous studies analyzed only some groups of the population or individual indicators, such as newly diagnosed patients, MDR-TB infections, the stability of solutions to systems of equations, and the basic reproduction number. To date, there is no mathematical model that comprehensively considers the epidemiologic characteristics of pulmonary tuberculosis among the whole population in China over the past decade or longer.

In this study, we developed a discrete dynamic model. The parameters were estimated from literature searches and data released by the government. We assessed trends in indicators that analyze the time and population distribution trends of pulmonary tuberculosis in China from 2004 to 2015. Additionally, we evaluated the implementation status of the national pulmonary tuberculosis prevention and control plan. Our study is of great significance because it provides a deeper understanding of the epidemiological characteristics and can help in developing more effective prevention and control policies.

## Methods

### Data sources

The data used in this study, which were national data from 2004 to 2015, came from the China Center for Public Health Science Data repository [13]. The data included the number of newly diagnosed pulmonary tuberculosis cases per month and the proportion of infectious cases (positive sputum smear and positive sputum culture) among the monthly newly diagnosed cases (Table a1).

### Model construction

**Fig. 1:**
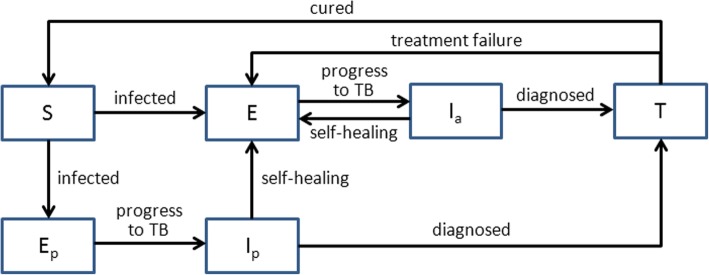
Block diagram of pulmonary tuberculosis transmission. Legend: *S* represents susceptible individuals who have not been infected with *M. tuberculosis*; *E* represents individuals who are infected with *M. tuberculosis* but have not developed the disease; *I*_*a*_ represents patients with secondary pulmonary tuberculosis who have symptoms but have not yet received treatment; *E*_*p*_ represents infected individuals who are in the incubation period; *I*_*p*_ represents primary tuberculosis patients who have symptoms but have not yet received treatment; and *T* represents patients who are being treated

Most people who do not have pulmonary tuberculosis at any given time can be classified into two states: *S*_,_ i.e., susceptible individuals who are not infected with *M. tuberculosis* (Fig. 1), and *E*_,_ i.e., those who have been infected with *M. tuberculosis* but are in the latent state and have not become sick. The source of infection is mainly infectious cases among secondary tuberculosis patients who have not yet been treated (*I*_*a*_) [14]. Patients in status *I*_*a*_ excrete *M. tuberculosis* into the air when coughing and spitting, either infecting individuals in state *S*, who then develop primary tuberculosis after an incubation period (the interval of time from the infection to the onset of symptoms), or causing those in state *E* to develop secondary pulmonary tuberculosis due to re-exposure to *M. tuberculosis* [14].

When individuals in state *S* become infected, a small proportion of infected people do not show clinical signs and transition to state *E*. Most infected people undergo an incubation period before they develop clinical symptoms, denoted by state *E*_*p*_ [15]. After the incubation period, individuals in state *E*_*p*_ develop fever, cough, chest pain and other symptoms, and transition to the state, *I*_*p*_. Since newborns are susceptible to tuberculosis, newborns in all populations are born in state *S*.

The state *I*_*a*_ includes two kinds of patients: those from state *E* who experience disease onset due to the recurrence of latent *M. tuberculosis* triggered by reduced immunity and those who transition from state *E* after interaction with individuals in state *I*_*a*_ and re-exposure to *M. tuberculosis* [14]. Some *I*_*a*_ and *I*_*p*_ patients visit medical institutions or pulmonary tuberculosis prevention and treatment institutions for diagnosis and treatment, while others abandon treatment because of mild symptoms or economic difficulties [4]. Some untreated patients will experience spontaneous recovery after a period of time. Since *M. tuberculosis* in the body is difficult to completely eradicate via spontaneous recovery, this group transitions to state *E* [14]. Other untreated patients will exhibit worsening symptoms and subsequently die.

State *T* represents patients from *I*_*a*_ and *I*_*p*_ who are undergoing treatment. Patients in state *T* can be infected by either MDR-TB or non-MDR-TB (including drug-sensitive tuberculosis and mono-resistant tuberculosis, which is resistant to any type of first-line drug) [16]. We modeled the two infections simultaneously by assuming a longer treatment duration and lower treatment completion rate for MDR-TB patients [17]. After a period of intensive treatment and consolidating treatment, a small number of patients will die due to severe illness, whereas the symptoms in the vast majority of patients will resolve. Some of the patients whose symptoms resolve will be cured (i.e., the *M. tuberculosis* bacteria in the body are completely eradicated), and these patients convert to state *S*. The other patients in whom *M. tuberculosis* is not completely eliminated due to intermittent adherence to treatment or inability to complete the entire course of chemotherapy transition to state *E*.

In this study, we distinguished between MDR-TB and non-MDR-TB when analyzing treatment processes and outcomes. However, certain relevant parameters associated with other considerations, such as rate of reactivation, incubation period, and proportion of patients seeking treatment, among others, have not been proven to differ for these two types of tuberculosis based on available documents. Fortunately, approximations of parameters for both MDR-TB and non-MDR-TB can be directly or indirectly acquired from literature sources (Table 1).

**Table 1:** Description and values of model parameters

Name	Definition	Mean and 95% CI	Source
*r*_*s*_	Rate at which patients complete treatment	0.125 (0.111, 0.1667)	[14]
*μ*_*b*_	Natural birth rate	0.001	[27]
*μ*_*d*_	Natural death rate	0.00059	[27]
*α*	Proportion of patients infected with non-MDR-TB	0.898 (0.878–0.9164)	[16]
*q*_1_	Cure rate of patients infected with non-MDR-TB	94%	[1]
*q*_2_	Cure rate of patients infected with MDR-TB	57%	[28]
*θ*	Rate at which an infected person develops disease due to reduced immunity and subsequent proliferation of *M. tuberculosis* already in the body	0.00026 (0.00013, 0.00039)	[4, 27]
*δ*	Rate of spontaneous recovery in patients with primary pulmonary tuberculosis	0.5	[1]
*r*_1_	Rate of treatment completion in patients infected with non-MDR-TB	0.1667	[17]
*τ*	Rate at which an infected person in the incubation period converts to primary pulmonary tuberculosis	0.206 (0.1542, 0.2765)	[15]
*r*_2_	Rate of treatment completion in patients infected with MDR-TB	0.05	[17]
*λ*	Proportion of susceptible individuals who have been infected with *M. tuberculosis*, but show no symptoms	0.19	[15]
*ν*	Proportion of pulmonary tuberculosis patients seeking treatment	0.472	[4]
*κ*_*s*_	Rate of *M. tuberculosis* is completely eliminated	0.939 (0.928, 0.947)	[29]
*κ*_*f*_	Rate of *M. tuberculosis* is not completely eliminated	0.014 (0.011, 0.019)	[29]
*d*_*f*_	Rate of death from pulmonary tuberculosis without treatment	0.019 (0.015, 0.024)	[30]
*d*_*h*_	Rate of spontaneous recovery in patients with secondary pulmonary tuberculosis	0.0069	[14]
*d*_*t*_	Rate of death of pulmonary tuberculosis patients after treatment	0.013 (0.011–0.016)	[29]
*p*(*t*)	Proportion of patients with infectious pulmonary tuberculosis at time *t*	Table a1	[13]
*f*()	Probability density distribution function of the average infectious period	Gamma distribution shape = 6.612; scale = 2.755 Weibull distribution shape = 2.782; scale = 2.696 log-normal distribution mean = 0.805; SD = 0.375	[18]

The infectious period (the interval of time from symptom onset to diagnosis) of an infected person is approximately 72 days on average (SD = 28) [18]. There is no published literature on the probability distribution of the infectious period. Based on our experience, we made the following assumptions. There are relatively few patients who undergo diagnosis and treatment soon after the onset of illness or after an extended period. Most patients seek treatment within a time interval near the average infectious period. We therefore assumed that the infectious period conforms to three common distributions: the Gamma distribution, the Weibull distribution, and the log-normal distribution [19]. We calculated the model output under these three distribution assumptions.

### Model fitting

**Fig. 2:**
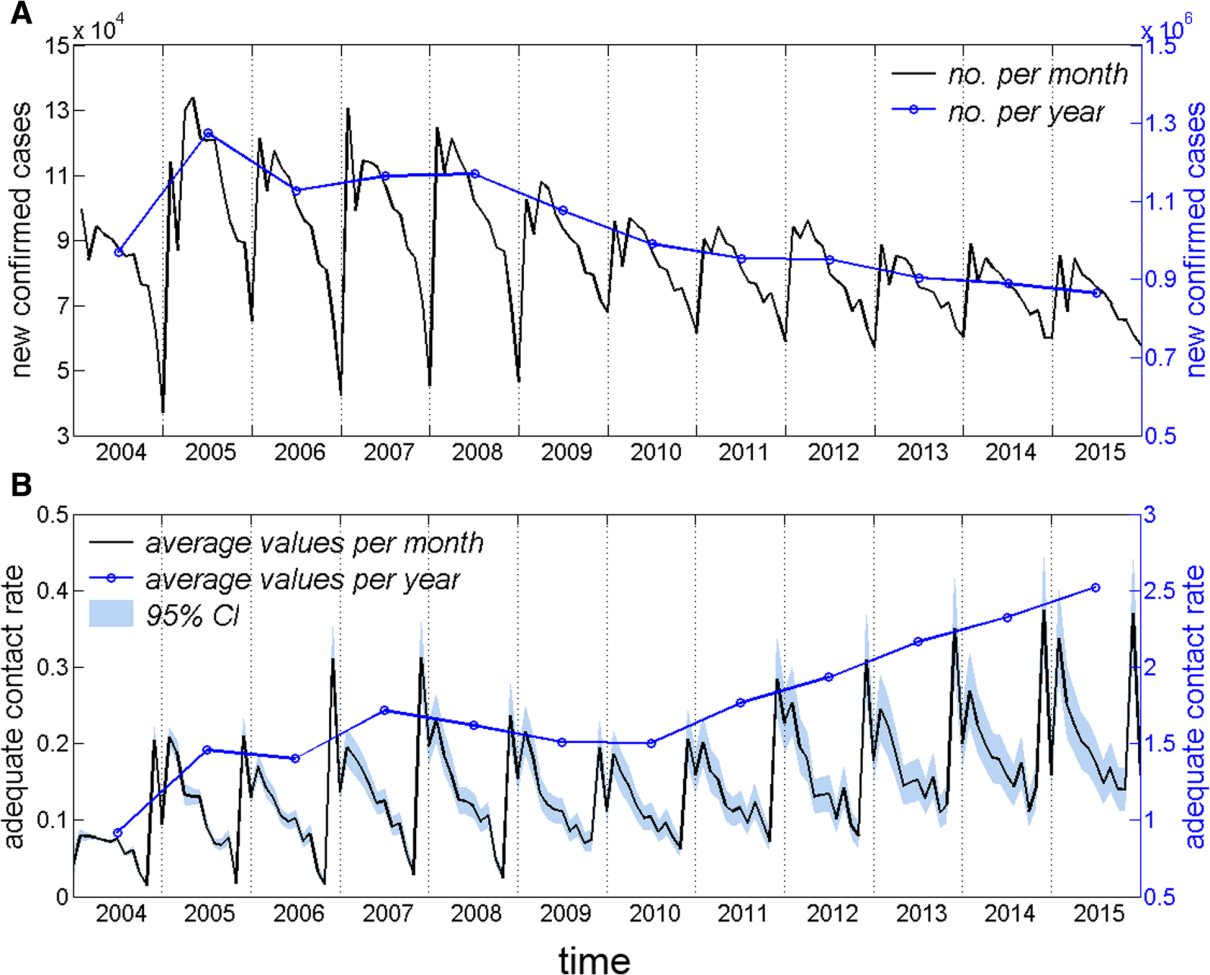
Trends in newly diagnosed pulmonary tuberculosis cases and adequate contact rate over time. Legend: **a** Distribution of newly diagnosed pulmonary tuberculosis cases over time. **b** Distribution of the adequate contact rate over time under three distribution hypotheses. The solid black line represents the mean per month, and the blue area represents the 95% PI, which is measured by the ordinate on the left; the blue line measured by the ordinate on the right represents the mean per year

The data fitted to the model are official data for newly diagnosed patients from January 2004 to December 2015 in mainland China, which are discrete and show obvious seasonal fluctuations, as indicated in Fig. 2a. First, we chose to develop a discrete dynamical model such that the adequate contact rate, which is the only parameter to be estimated in the model, also has a discrete distribution over time. Second, the adequate contact rate over time was obtained by fitting the model output, the number of newly diagnosed cases in each month, to official published data (Fig. 2b). Finally, the values of adequate contact rate were introduced into the equations to obtain the target indicators.

In each iteration, we generate a random value for each parameter following normal distributions, which means and standard deviations (SD) are extracted from the literatures as shown in Table 1. After all the parameters are generated, they are incorporated into equation a1, and a set of values for the adequate contact rate is obtained by fitting the model output to the original data. After 500 iterations, the mean and 95% prediction interval (PI) at each time point are calculated.

### Epidemiological indicators

We chose 6 indicators to evaluate the epidemiological trend of pulmonary tuberculosis: adequate contact rate, prevalence (the proportion of China’s total population composed of untreated patients with pulmonary tuberculosis), infection rate (the proportion of China’s total population composed of persons who are infected with *M. tuberculosis* but have not yet become sick), the number of infectious patients in the community, the rate of new patients (the number of new patients per month), and the rate of new infections (the number of people infected with *M. tuberculosis* per month). The parameters of this model are listed in Table 1, and their positions are shown in equation a1.

### Sensitivity analyses

Partial rank correlation coefficients (PRCC) and Latin hypercube sampling (LHS) were used to conduct sensitivity analyses. PRCC-LHS is an efficient and reliable sampling-based sensitivity analysis method that provides a measure of monotonicity between a set of parameters and the model output after removing the linear effects of all parameters except the parameter of interest [20]. Each parameter interval (from 0.5 to 1.5 times the average value of the parameter) was divided into *N* smaller and equal intervals, and one sample was selected randomly from each interval [20, 21]. A standard coefficient denoting the correlation between the parameter and model output was calculated.

## Results

### Newly diagnosed cases and adequate contact rate

Using official data, we plotted the time distribution of newly diagnosed cases, as shown in Fig. 2a. This distribution is characterized by significant seasonal fluctuations. The epidemic cycle lasts 1 year. The number of diagnosed patients is highest in the first quarter of each year. After that, the number of patients continues to decrease and then reaches its lowest level at the end of the year. The year 2005 had the largest number of people with disease onset, totaling 1,275,121. After 2008, the number of people with disease onset decreased each year and reached as low as 864,015 by 2015; the average annual decline was approximately 4.2%. The adequate contact rate showed similar epidemiological patterns as the number of newly diagnosed cases, with obvious seasonal changes. One key difference is that the peak of the adequate contact rate appeared 2 months earlier than the peak of newly diagnosed cases, as shown in Fig. 2b. Contrary to the declining trend of newly diagnosed cases, the adequate contact rate gradually increased. It was lowest in 2004, with an average of 0.7/year, and highest in 2015, with an average of 2.5/year. The adequate contact rate has been increasing since 2010, with an average annual increase of 11.8%. Regardless of whether the infectious period was modeled using a gamma distribution, a Weibull distribution, or a log-normal distribution, the mean values and the 95% PIs of the adequate contact rate coincided.

### Prevalence, infection rate, and the number of infectious patients

**Fig. 3:**
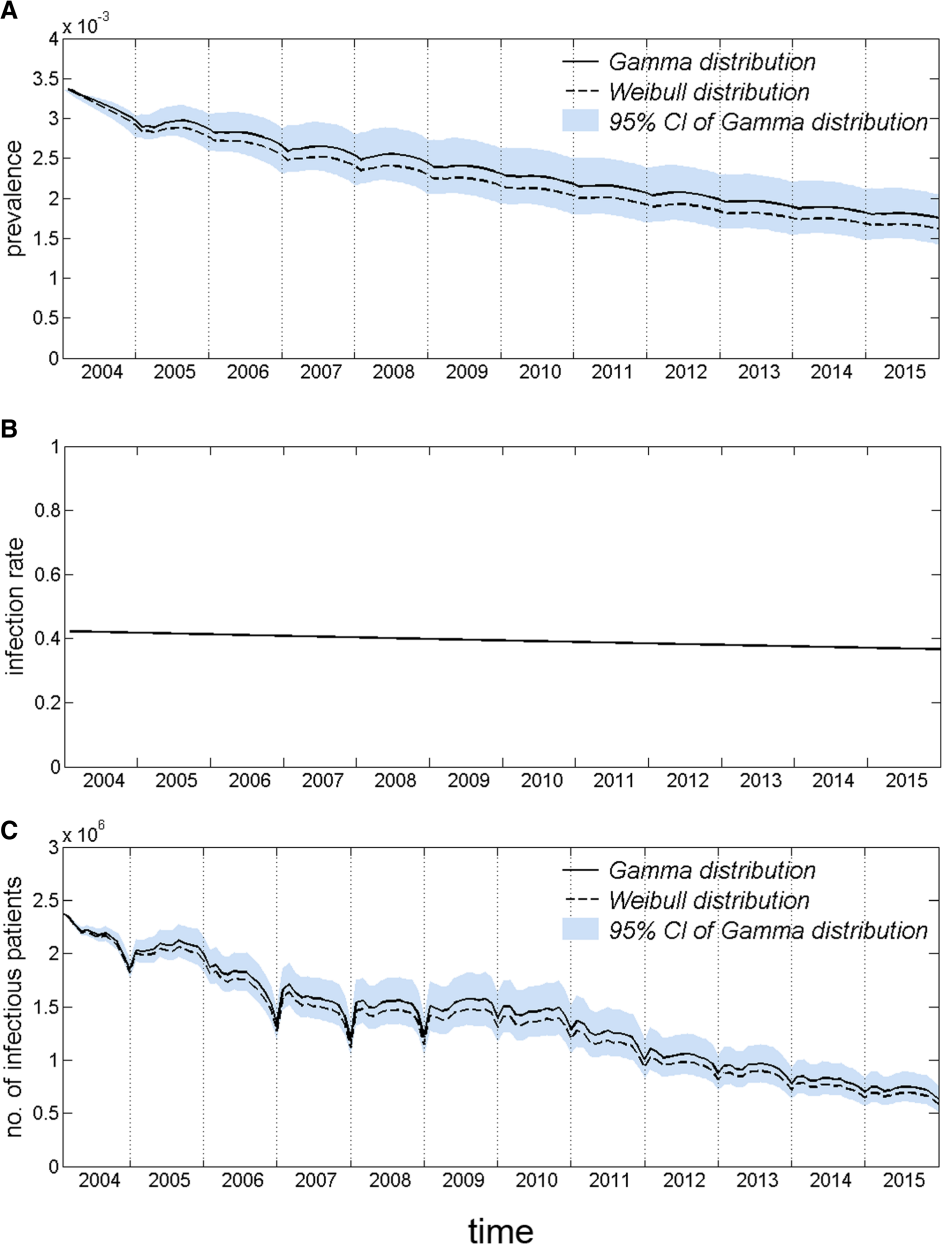
Trends in prevalence and infection rate in the total population and the number of infectious patients over time. Legend: **a** Prevalence in the total population over time; the solid black line indicates the mean prevalence under the gamma distribution, the dashed black line indicates the mean prevalence under the Weibull distribution and the log-normal distribution, and the blue area indicates the 95% PI of prevalence under the gamma distribution. **b** Infection rate in the total population over time. **c** The number of infectious patients over time

The prevalence in the total population decreased over time, as shown in Fig. 3a, with slight seasonal fluctuations. Regardless of the probability distribution used for the infectious period, the trends remained almost identical. The prevalence continued to decrease from 336/100,000 at the beginning of 2004 to 175/100,000 at the end of 2015, with an average annual drop of 5.3%. The infection rate in the total population showed a slight linear decrease, and there was essentially no fluctuation range. As shown in Fig. 3b, the infection rate decreased from 42.3% at the beginning of 2004 to 36.6% at the end of 2015, with an average annual decline of 1.2%. The number of infectious patients decreased from 2.38 million at the beginning of 2004 to 0.63 million at the end of 2015, with an average annual decline of 10.5%, as shown in Fig. 3c. Both the prevalence and the number of infectious patients were essentially identical under the Weibull and log-normal distributions, whereas the lines were slightly higher under the gamma distribution.

### Growth rates for new infections and new tuberculosis cases

**Fig. 4:**
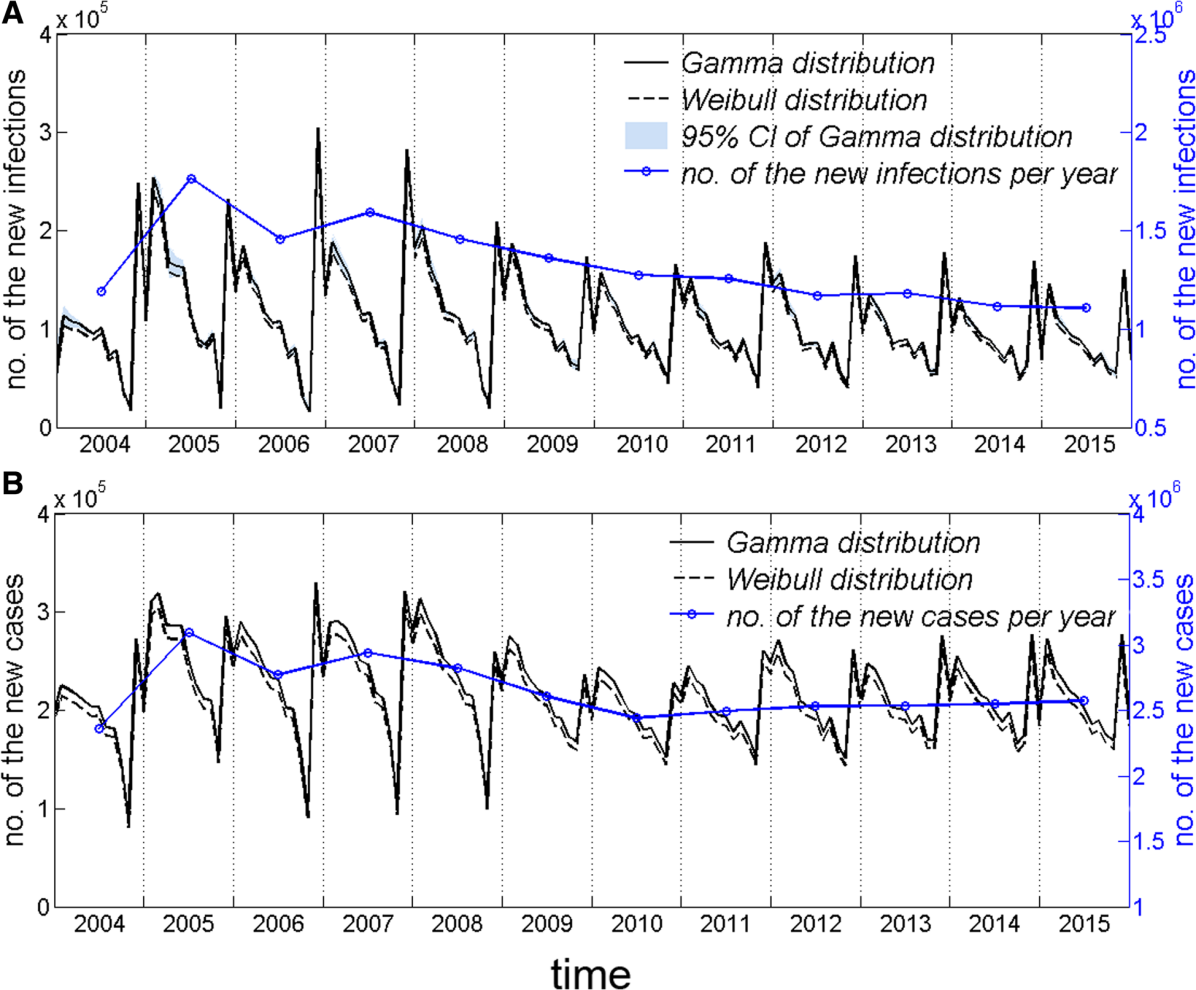
Trends in the growth rates of new infections and new cases. Legend: **a** Trends in the growth rate of new infections. The solid black line represents the mean number of new infections per month under the gamma distribution condition, the black dotted line represents the mean number of new infections per month under the Weibull distribution and the log-normal distribution condition, the blue area represents the 95% PI under the gamma distribution, which is measured by the ordinate on the left; the blue line measured by the ordinate on the right represents the mean per year; **b** Trends in growth rates of new cases

Both new infections and new cases showed obvious seasonal fluctuation (Fig. 4), and the trends over time were similar to those observed for the adequate contact rate. The growth rate of new infections (Fig. 4a) and new cases (Fig. 4b) under the gamma distribution was slightly faster than the rate obtained using the Weibull distribution, which was extraordinarily similar to the log-normal distribution. The growth rate of new pulmonary tuberculosis cases was generally higher than that of new infections, and both were relatively stable after 2010. The fluctuation range of the growth rate of new infections was very narrow, and there was almost no fluctuation in the growth rate of new cases. When the infectious period was modeled using the gamma distribution, there was an average increase of 1.1 × 10^6^ new infections and 2.5 × 10^6^ new cases in 2015.

### Sensitivity analyses

**Fig. 5:**
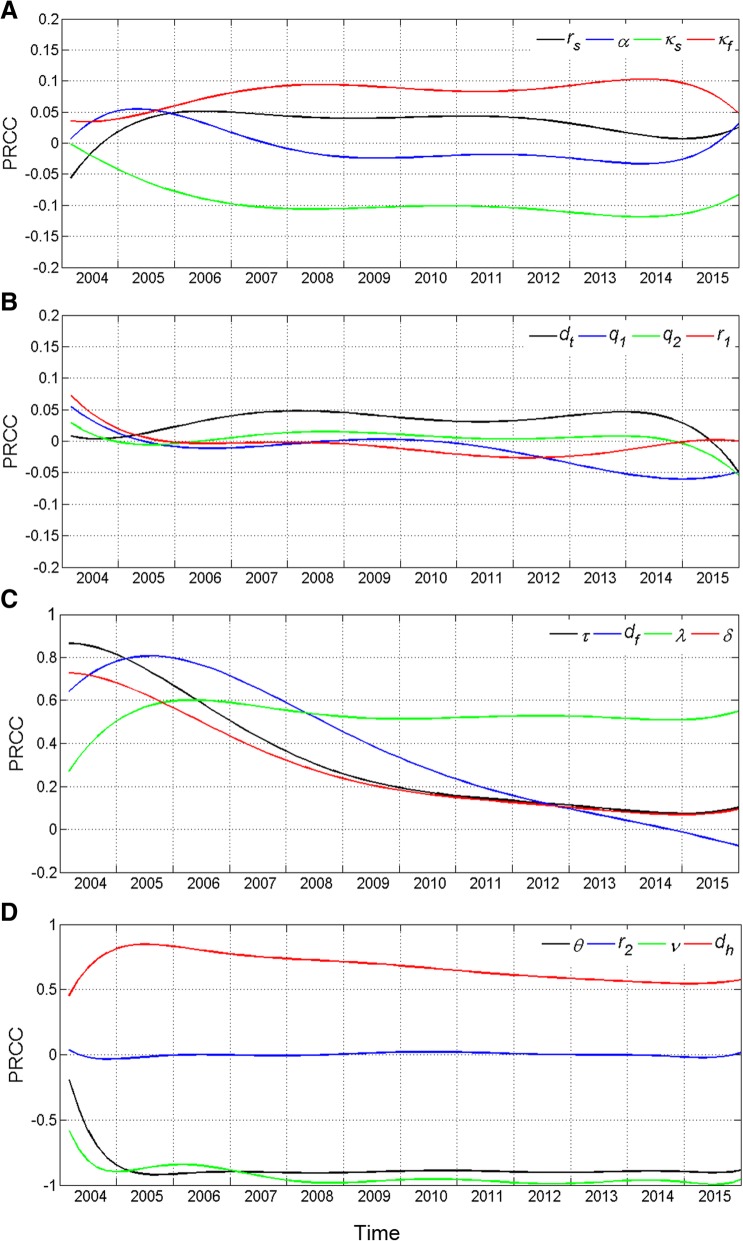
Sensitivity analysis of the continuous time series. Legend: Results of the sensitivity analysis including the following parameters: **a** *r*_*s*_, *α*, *κ*_*s*_, *κ*_*f*_; **b** *d*_*t*_, *q*_1_, *q*_2_, *r*_1_; **c** *τ*, *d*_*f*_, *λ*, *δ*; **d** *θ*, *r*_2_, *ν*, *d*_*h*_

In this study, sensitivity analyses were conducted with 16 parameters and a continuous time series for the population infection rate each month. We took *N* = 500 samples from a uniform distribution for each parameter range. PRCCs near − 1 or + 1 indicate that the parameter has a strong impact on the output, whereas those closer to 0 indicate less effect on the output result for that parameter (Fig. 5). The results reflected that *r*_*s*_, *α*, *κ*_*s*_, *κ*_*f*_, *d*_*t*_, *q*_1_, *q*_2_, *r*_1_, and *r*_2_ had less effect on the model outputs; *τ*, *d*_*f*_, *λ*, *δ*, and *d*_*h*_ had more effect; meanwhile, *θ* and *r*_2_ had the strongest effect. All analyses were conducted using MATLAB R2012a (The MathWorks, USA, 2012).

## Discussion

The number of newly diagnosed pulmonary tuberculosis patients and the adequate contact rate show seasonal fluctuations, as is seen in many respiratory infections such as influenza and measles [22–25]. The number of confirmed patients in February was slightly lower, probably due to a decrease in the number of hospital visits during the one-week Spring Festival holiday. Since the adequate contact rate is estimated based on official data, it was also slightly lower at the corresponding time in February, and this effect cannot be eliminated by the model. The peak in the adequate contact rate was observed in November, indicating that pulmonary tuberculosis is most contagious in autumn and winter. This is consistent with the results of previously published studies [9]. Therefore, we should focus on strengthening prevention and control in autumn and winter, and the government should increase publicity and education on pulmonary tuberculosis in this season, strengthen management measures, and encourage the public to pay attention to personal protection. The peak in the number of patients newly diagnosed with pulmonary tuberculosis lagged behind the peak in the adequate contact rate by 2 months. The reason is that when some infected persons are re-infected with *M. tuberculosis* and develop active tuberculosis, it takes an average of 72 days between disease onset and confirmation of the diagnosis. According to the algorithm described in the Additional file 1, the probability of being diagnosed is greatest at 2–3 months after symptom onset. The adequate contact rate has increased yearly since 2010. This may be due to increased population mobility due to urbanization in China, mutations of pathogenic bacteria leading to increased infectiousness and increased urban population density. Our findings suggest that the efficiency of pulmonary tuberculosis transmission has actually increased.

According to the results of the Fourth National Survey of Pulmonary Tuberculosis Epidemiology, the prevalence of active pulmonary tuberculosis in China in 2000 was 367 per 100,000 [26]. Since the prevalence is decreasing every year, we adjusted the prevalence of active pulmonary tuberculosis in early 2004 to 340 per 100,000 according to the annual rate of decline. Based on this calculation, the annual rate of decline in active pulmonary tuberculosis in 2004–2015 was 5.3%. Comparison of the prevalence in 1990 with that in 2000 showed an annual decline of 5.4% [26], indicating that the model estimation results are in line with the actual survey results. The Fifth National Survey of Pulmonary Tuberculosis Epidemiology conducted in 2010 did not include people under the age of 15 years. Therefore, since 2000, no actual survey data have been available to compare with the prevalence estimated by the model. The overall rate of *M. tuberculosis* infection has also been decreasing, but the rate of decline is relatively slow. The fluctuation range of the infection rate is very narrow, indicating that changes in the model parameters have little effect on the model. Based on the decline in the prevalence and infection rate, it can be clearly determined that the epidemic of tuberculosis in China is being effectively controlled and that comprehensive prevention and control measures implemented by the government have achieved significant results.

The number of newly diagnosed patients released by the government does not represent the actual numbers of new patients and new infections. The latter two values reflect the severity of the pulmonary tuberculosis epidemic in real time and are important criteria for measuring prevalence. However, they cannot be directly observed. Using the mathematical model, we found that these indicators also exhibited significant seasonal changes, and trends over time were similar to those observed for the adequate contact rate. This can be explained by the fact that when the adequate contact rate is high, pulmonary tuberculosis spreads rapidly, which leads to a corresponding increase in the growth rate of infected persons. In addition, the growth rate of new patients is also affected by the adequate contact rate because as the adequate contact rate increases, more infected people in state *E* develop secondary pulmonary tuberculosis due to increased re-exposure to *M. tuberculosis*. A high adequate contact rate also increases the number of individuals in state *E*_*p*_, leading to an increase in the number of new primary pulmonary tuberculosis patients.

We distinguished MDR-TB and non-MDR-TB in our analyses. The treatment cycle and cure rate of the two types of *M. tuberculosis* are different. To ensure that the model’s analysis of pulmonary tuberculosis treatment is as realistic as possible, we considered MDR-TB and non-MDR-TB separately. The mortality associated with pulmonary tuberculosis is not considered in the model. We made this choice because the mortality rate of patients seeking initial treatment and that of patients seeking repeat treatment are different, and the mortality rates of MDR-TB and non-MDR-TB infection are also different. There is currently not enough literature to determine the parameters needed to assess the mortality rate of pulmonary tuberculosis.

Parameter *κ*_*s*_ is negatively correlated with the infection rate because the sum of the cure rate and the treatment failure rate is less than 1. A higher cure rate results in a lower treatment failure rate, and fewer patients convert to state *E* after treatment. Similarly, *κ*_*f*_ is positively correlated with the infection rate. *τ* is positively correlated with the infection rate such that a larger increase in the number of patients with primary pulmonary tuberculosis results in a greater number of conversions to state *E* after spontaneous recovery. The positive correlation effect of *d*_*f*_ and the infection rate gradually decreases because as more patients with secondary tuberculosis die and survival decreases, fewer people become infected due to exposure to *M. tuberculosis*. Thus, the number of individuals in state *E* increases, and as time goes by, this effect gradually diminishes. *λ* is positively correlated with the infection rate because as the number of asymptomatic infections grows faster, the number of individuals in state *E* increases. *δ* is positively correlated with the infection rate because when the number of patients who undergo spontaneous recovery from primary pulmonary tuberculosis is larger, the number of individuals in state *E* is greater. *θ* is negatively correlated with the infection rate because as the number of infected individuals who experience reactivation due to reduced immunity increases, the number of individuals in state *E* decreases. *ν* is negatively correlated with the infection rate because as the number of patients seeking treatment increases, the number of patients undergoing spontaneous recovery and entering state *E* decreases, and the reduction in the number of infectious patients also reduces the number of new infections. *d*_*h*_ is positively correlated with the infection rate because as the number of spontaneous recovery individuals increases, the number of individuals in state *E* increases. The PRCC of other parameters is near 0, indicating that their influence on the infection rate is very weak.

MDR-TB and non-MDR-TB should be distinguished throughout all stages because both forms of tuberculosis are found among infected individuals and patients. However, certain relevant parameters, such as the rate of reactivation and the incubation period, among others, have not been proven to differ between MDR-TB and non-MDR-TB based on available reports. We are restricted to using approximations of these parameters based on literature sources to integratively represent their characteristics. This limitation may have affected the accuracy of the results to a certain extent.

Since most of the parameters in the model are obtained directly from literature searches, they are all set at a fixed value. However, many parameters change with time, and we cannot accurately obtain the values of these changing parameters, which affects the model’s estimated results to some extent. Our model does not cover all situations that manifest in reality. For example, we did not consider the immune effects of the Bacille Calmette-Guerin vaccine, tuberculin test screening, centralized diagnosis and treatment for military, student and other special populations, as well as the differences between age groups, because we did not have enough data to include these parameters in a model. Nevertheless, the model estimates are consistent with the national surveys of pulmonary tuberculosis and the conclusions of available studies, indicating that the model faithfully reflects the epidemiological patterns of pulmonary tuberculosis in China since 2004.

## Conclusions

Therefore, based on the current research, we believe that the prevalence and infection rates of tuberculosis in China have decreased and that the number of new infections and new patients per year remains relatively stable. However, infectiousness is gradually increasing, which represents a new challenge for the health system.

## Additional file

Additional file 1: 1. Construction of the model; 2. Raw data. 1. According to the epidemic characters of tuberculosis in China, we established a mathematical model, and elaborated on the construction of the model and the process of mathematical derivation; 2. Raw data that are essential for calculation of the model from the year of 2004 to 2015 are publicly available. (PDF 145 kb)

## Abbreviations

CI: Confidence interval
LHS: Latin hypercube sampling
*M. tuberculosis*: *Mycobacterium tuberculosis*
MDR-TB: Multidrug-resistant tuberculosis
PI: Prediction interval
PRCC: Partial rank correlation coefficients
SD: Standard deviation
