# Supplementary material for: Epidemiological characteristics of pulmonary tuberculosis in mainland China from 2004 to 2015: a model-based analysis
Source: BMC Public Health. 2019 Feb 21;19:219. doi: 10.1186/s12889-019-6544-4 (PMC6383277; doi:10.1186/s12889-019-6544-4)
Supplement: Supplementary file 1 — 1. Construction of the model; 2. Raw data. 1. According to the epidemic characters of tuberculosis in China, we established a mathematical model, and elaborated on the construction of the model and the process of mathematical derivation; 2. Raw data that are essential for calculation of the model from the year of 2004 to 2015 are publicly available. (PDF 145 kb) [file 12889_2019_6544_MOESM1_ESM.pdf]

Additional file

We describe in detail the structure of the model below.

Construction of the model

Based on the epidemiologic characteristics of pulmonary tuberculosis described in Figure 1, we devised the following system of discrete difference equations:

$$
\begin{aligned}
S(t+1) &= (1-\mu_d)S(t) + \mu_b N(t) - \beta(t)p(t)I_a(t)S(t)/N(t) + \kappa_s \alpha r_1 q_1 T(t) + \kappa_s (1-\alpha) r_2 q_2 T(t) \\
E(t+1) &= (1-\mu_d)E(t) + \lambda\beta(t)p(t)I_a(t)S(t)/N(t) + \delta I_p(t) + d_h I_a(t) + \kappa_f \alpha r_1 q_1 T(t) + \kappa_f (1-\alpha) r_2 q_2 T(t) \\
&\qquad - \theta E(t) - \beta(t)p(t)I_a(t)E(t)/N(t) \\
I_a(t+1) &= (1-\mu_d)I_a(t) + \beta(t)p(t)I_a(t)E(t)/N(t) + \theta E(t) - Q_a(t) - d_f I_a(t) - d_h I_a(t) \\
E_p(t+1) &= (1-\mu_d)E_p(t) + (1-\lambda)\beta(t)p(t)I_a(t)S(t)/N(t) - \tau E_p(t) \\
I_p(t+1) &= (1-\mu_d)I_p(t) + \tau E_p(t) - \delta I_p(t) - Q_p(t) \\
T(t+1) &= (1-\mu_d)T(t) + Q_a(t) + Q_p(t) - (\kappa_f + \kappa_s)\alpha r_1 q_1 T(t) - (\kappa_f + \kappa_s)(1-\alpha) r_2 q_2 T(t) - d_t r_s T(t) \\
N(t) &= S(t) + E(t) + I_a(t) + E_p(t) + I_p(t) + T(t)
\end{aligned}
$$

(a1)

$$
\begin{aligned}
Q_a(t) &= \nu \int_{t-1}^{t} \sum_{k=o}^{t-1} \Delta^+ I_a(k) f(s-k+0.5) \exp\{-(d_f + d_h)(s-k+0.5)\} ds \\
Q_p(t) &= \nu \int_{t-1}^{t} \sum_{k=o}^{t-1} \Delta^+ I_p(k) f(s-k+0.5) \exp\{-(d_f + d_h)(s-k+0.5)\} ds \\
\Delta^+ I_a(t) &= p(t)\beta(t)I_a(t)E(t)/N(t) + \theta E(t) \\
\Delta^+ I_p(t) &= \tau E_p(t)
\end{aligned}
\qquad \text{(a2)}
$$

$S(t)$ represents individuals who are susceptible to *M. tuberculosis* and not infected with *M. tuberculosis* at time t ($t = 0,1,2,\cdots,144$) (equation a1). The rate of increase includes three components: one is $\mu_b N(t)$, which denotes the growth rate of newborns in China at time t, as they have no immunity to tuberculosis and are considered to be susceptible; the remaining components are $\kappa_s \alpha r_1 q_1 T(t)$ and

$\kappa_s(1-\alpha)r_2 q_2 T(t)$, which represent the rates of complete cure (the *M. tuberculosis* bacteria in the body are completely eliminated) after treatment in patients infected with non-MDR-TB and MDR-TB, respectively. The rate of reduction includes two components: one is $\mu_d S(t)$, which represents the rate at which the susceptible population is reduced by natural death at time t; the other is $\beta(t)p(t)I_a(t)S(t) / N(t)$, which represents the rate at which a susceptible person is infected by *M. tuberculosis* at time t. $\beta(t)$ represents the adequate contact rate. $p(t)I_a(t)$ represents the number of infectious patients (positive sputum smear or sputum culture). $p(t)$ represents the proportion of infectious patients among newly diagnosed pulmonary tuberculosis patients, and its value varies with time (Table a1). We used $p(t)$ to replace the proportion of infectious patients in $I_a(t)$.

$E(t)$ denotes infected individuals who have been infected with *M. tuberculosis* but who are in the incubation period and have not yet developed active disease at time t. The rate of growth includes five components: $\lambda p(t)\beta(t)I_a(t)S(t) / N(t)$ represents the growth rate of infected individuals who do not develop active disease. This occurs because a small number of susceptible people do not show symptoms after infection, and this group of infected people transition to state E. $\delta I_p(t)$ represents the rate of spontaneous recovery without treatment in patients with primary pulmonary tuberculosis. Because *M. tuberculosis* is difficult to completely eradicate due to spontaneous recovery, these individuals convert to state E. $\kappa_f \alpha r_1 q_1 T(t)$ and $\kappa_f(1-\alpha)r_2 q_2 T(t)$ represent the rates of treatment failure in patients with non-MDR-TB and MDR-TB infection, respectively. Treatment failure indicates that a patient's

symptoms and infectiousness resolve but the patient is not cured; the *M. tuberculosis* bacteria in the body are not completely removed, and the patient transitions to state E; $d_h I_a(t)$ represents the rate of spontaneous recovery of patients with secondary tuberculosis. The rate of reduction of $E(t)$ includes three components: $\mu_d E(t)$ represents the rate of natural death in state $E(t)$; $\theta E(t)$ represents the rate at which an infected person develops disease due to reduced immunity and subsequent proliferation of *M. tuberculosis* already in the body; and $\beta(t)p(t)I_a(t)E(t) / N(t)$ represents the rate at which an infected person develops disease due to contact with a contagious secondary tuberculosis patient.

$I_a(t)$ represents the number of patients with secondary pulmonary tuberculosis who have not been diagnosed at time t. Its rate of increase depends on two components: $\beta(t)p(t)I_a(t)E(t) / N(t)$ and $\theta E(t)$. The rate of reduction depends on four components: the rate of natural death $\mu_d I_a(t)$; the rate of diagnosis $Q_a(t)$, which is computed as shown in equation a2; and $d_f I_a(t)$ and $d_h I_a(t)$, which represent the rates of death and spontaneous recovery, respectively, assuming that the distributions of death and spontaneous recovery are exponential such that d_f and d_h are constants.

$E_p(t)$ denotes the number of infected people in the incubation period at time t. The growth rate is $(1-\lambda)\beta(t)p(t)I_a(t)S(t) / N(t)$, indicating the growth rate of infected persons with symptoms. In addition to natural deaths, the rate of decline also includes the rate of conversion from the incubation period to primary pulmonary tuberculosis $\tau E_p(t)$.

$I_p(t)$ represents the number of primary pulmonary tuberculosis patients who have not been treated at time t. The growth rate is $\tau E_p(t)$. In addition to natural deaths, the rate of reduction includes the rate of self-healing $\delta I_p(t)$ as well as $Q_p(t)$, which represents the rate at which patients with primary tuberculosis are diagnosed.

$T(t)$ indicates the number of pulmonary tuberculosis patients being treated at time t. The rate of increase consists of the rates of diagnosis of patients with secondary and primary tuberculosis, $Q_a(t)$ and $Q_p(t)$. In addition to natural death, the rate of decrease also includes $(\kappa_f + \kappa_s)\alpha r_1 q_1 T(t)$ and $(\kappa_f + \kappa_s)(1-\alpha) r_2 q_2 T(t)$, which represent the rates at which symptoms disappear after a complete course of treatment in patients with non-MDR-TB and MDR-TB, respectively; $d_t r_s T(t)$ represents the rate at which patients die after a complete course of treatment. Mortality information for treated patients with non-MDR-TB and MDR-TB is unavailable in the relevant literature. Instead, we eventually determine the mortality of treated patients of both types, d_t, and the rate of treatment completion, r_s. Therefore, we must discuss mortality in both cases.

$Q_a(t)$ and $Q_p(t)$ represent the rates of diagnosis of patients with secondary and primary pulmonary tuberculosis in month t, respectively (equation a2). We assume that $I_a(t)$ and $I_p(t)$ include new secondary and primary tuberculosis patients over several months before month t. Each month, a proportion ν of the newly diagnosed patients choose to receive treatment; the remaining patients ($1-\nu$) do not pursue treatment. Only patients who choose to receive treatment are counted as diagnosed cases. As shown in equation a2, any month before month t is represented by k (

$o \leq k \leq t-1$, with o indicating that a certain month occurs long before month t). The rates of increase in states I_a and I_p in the k th month and are represented by $\Delta^+ I_a(k)$ and $\Delta^+ I_p(k)$, respectively, with $f(\bullet)$ representing the probability density function from onset to the time of diagnosis. We assume that the density function follows one of three possible distributions: a gamma distribution, a Weibull distribution, or a log-normal distribution.

$\nu \sum_{k=o}^{t-1} \Delta^+ I_a(k) f(s-k+0.5) \exp\{-(d_f + d_h)(s-k+0.5)\}$ represents the number of secondary pulmonary tuberculosis cases that occur before the t th month and are diagnosed at s ($t-1 \leq s \leq t$). The number of patients with secondary pulmonary tuberculosis diagnosed in the t th month was obtained by integrating s in the t th month. Because the equation is discrete, 0.5 means that the monthly increase in the number of patients is adjusted to the middle of the current month. According to our calculations, the majority of cases diagnosed in any month is mostly from new infections occurring in the preceding 5 months.

Setting initial values

According to the literature, approximately 550 million people were infected with *M. tuberculosis* in China in 2004 [1]. According to the fifth national census in 2000, the country has a total population of approximately 1.24 billion [2]. From these data, we obtained the initial values of S and E. The initial values of I_a, I_p, E_p, and T are estimated based on the solution when the equation is at equilibrium and estimated based on the total population prevalence in the 4th national pulmonary tuberculosis survey in 2000.

Epidemiological indicators of the model outputs

Trends in newly diagnosed pulmonary tuberculosis cases come from the raw data. Trends in adequate contact rate over time are obtained by fitting the model with the published number of newly diagnosed pulmonary tuberculosis cases. Trends in prevalence and infection rate in the total population are expressed as $(I_a(t) + I_p(t)) / N(t)$ and $E(t) / N(t)$ respectively. Trends in the number of infectious patients over time are expressed as $p(t)I_a(t)$. Trends in the growth rates of new infections and new cases are expressed as $\beta(t)p(t)I_a(t)S(t) / N(t)$ and $\beta(t)p(t)I_a(t)E(t) / N(t) + \theta E(t) + \tau E_p(t)$ respectively. The meanings of each formula have been explained previously.

Table a1. Raw data

Time	Number of patients with positive sputum culture	Number of patients with positive sputum smear	Number of patients with negative sputum	Number of patients not tested	Ratio of infectious patients	Total number of patients
Jan 2004	103	37803	28711	32849	0.569	99466
Feb 2004	109	31324	24064	28659	0.566	84156
Mar 2004	106	34638	27662	31954	0.557	94360
Apr 2004	145	32411	26891	32497	0.548	91944
May 2004	140	32834	26520	30885	0.554	90379
Jun 2004	123	31922	25831	29785	0.554	87661

Jul 2004	132	31315	25323	28347	0.554	85117
Aug 2004	136	31850	24691	29183	0.564	85860
Sep 2004	137	28173	22339	26037	0.559	76686
Oct 2004	94	27402	22075	26253	0.555	75824
Nov 2004	77	20134	17977	23381	0.529	61569
Dec 2004	40	9695	9722	17800	0.500	37257
Jan 2005	130	47567	37090	29407	0.563	114194
Feb 2005	101	35990	28895	22126	0.555	87112
Mar 2005	156	56228	44007	29743	0.562	130134
Apr 2005	167	57990	45607	30160	0.560	133924
May 2005	159	52877	39864	28426	0.571	121326
Jun 2005	138	52941	40539	27047	0.567	120665
Jul 2005	138	52941	40539	27047	0.567	120665
Aug 2005	153	47435	34322	25066	0.581	106976
Sep 2005	114	42640	31158	21829	0.578	95741
Oct 2005	122	39048	28610	22021	0.578	89801
Nov 2005	124	39188	28444	21620	0.580	89376
Dec 2005	79	26349	20308	18471	0.565	65207
Jan 2006	124	53121	45341	22849	0.540	121435
Feb 2006	125	45322	38498	21324	0.541	105269
Mar 2006	153	47588	43701	26135	0.522	117577
Apr 2006	176	44562	41981	25311	0.516	112030
May 2006	140	44293	39895	24855	0.527	109183
Jun 2006	145	40656	36875	23384	0.525	101060

Jul 2006	165	39056	34988	22017	0.529	96226
Aug 2006	145	36989	34856	22386	0.516	94376
Sep 2006	144	31955	31317	19595	0.506	83011
Oct 2006	149	29336	31130	20308	0.486	80923
Nov 2006	90	20686	24307	18999	0.461	64082
Dec 2006	49	9386	13827	19137	0.406	42399
Jan 2007	169	54468	50694	25406	0.519	130737
Feb 2007	120	41935	38340	18879	0.523	99274
Mar 2007	157	45043	45382	23946	0.499	114528
Apr 2007	153	43126	46156	24512	0.484	113947
May 2007	183	42422	44933	24836	0.487	112374
Jun 2007	140	39567	42830	24194	0.481	106731
Jul 2007	152	36668	39627	23421	0.482	99868
Aug 2007	158	35694	39049	23159	0.479	98060
Sep 2007	191	31787	34878	20651	0.478	87507
Oct 2007	197	29649	33367	21379	0.472	84592
Nov 2007	141	21982	27513	21300	0.446	70936
Dec 2007	66	8653	14449	22237	0.376	45405
Jan 2008	230	50247	50245	23903	0.501	124625
Feb 2008	170	44510	44814	21152	0.499	110646
Mar 2008	190	45341	49927	25804	0.477	121262
Apr 2008	206	42734	47950	24372	0.472	115262
May 2008	255	42086	44862	23630	0.486	110833
Jun 2008	191	39235	40762	21756	0.492	101944

Jul 2008	155	37902	39083	21748	0.493	98888
Aug 2008	176	36811	37216	21151	0.498	95354
Sep 2008	159	33716	34529	19412	0.495	87816
Oct 2008	160	32699	33514	20311	0.495	86684
Nov 2008	115	23488	26650	19299	0.470	69552
Dec 2008	61	9945	14887	21781	0.402	46674
Jan 2009	101	46112	44675	11762	0.508	102650
Feb 2009	91	39784	40331	11700	0.497	91906
Mar 2009	87	46477	48427	12859	0.490	107850
Apr 2009	104	45902	47328	12455	0.493	105789
May 2009	94	43852	41957	11787	0.512	97690
Jun 2009	104	42518	39554	11195	0.519	93371
Jul 2009	91	41787	37568	10941	0.527	90387
Aug 2009	100	40956	36494	10786	0.529	88336
Sep 2009	98	37188	33242	9513	0.529	80041
Oct 2009	75	37404	32226	9769	0.538	79474
Nov 2009	80	32667	29281	9548	0.528	71576
Dec 2009	99	25729	27563	14477	0.484	67868
Jan 2010	113	46027	41432	8445	0.527	96017
Feb 2010	75	39537	35789	6526	0.525	81927
Mar 2010	144	44003	44080	8661	0.500	96888
Apr 2010	144	42584	43280	8475	0.497	94483
May 2010	143	42559	41222	8736	0.509	92660
Jun 2010	175	40129	38704	7787	0.510	86795

Jul 2010	154	38230	35961	7543	0.516	81888
Aug 2010	136	38335	34559	7784	0.527	80814
Sep 2010	135	35231	31986	7112	0.525	74464
Oct 2010	134	36246	31245	7618	0.538	75243
Nov 2010	153	31048	29452	7947	0.514	68600
Dec 2010	111	22748	25849	12863	0.469	61571
Jan 2011	140	42120	41379	6831	0.505	90470
Feb 2011	149	39523	40357	6070	0.496	86099
Mar 2011	154	40226	46714	7093	0.464	94187
Apr 2011	166	37055	44848	7007	0.454	89076
May 2011	167	37233	43548	6837	0.462	87785
Jun 2011	166	34992	39690	6392	0.470	81240
Jul 2011	145	32884	38172	6121	0.464	77322
Aug 2011	164	32629	37372	6363	0.467	76528
Sep 2011	140	29947	34888	5947	0.463	70922
Oct 2011	175	30632	36175	6741	0.460	73723
Nov 2011	155	25167	33875	7721	0.428	66918
Dec 2011	123	17415	27981	13486	0.385	59005
Jan 2012	179	37588	50001	6226	0.430	93994
Feb 2012	181	34643	49206	6696	0.414	90726
Mar 2012	228	34606	53807	7517	0.393	96158
Apr 2012	181	32707	50534	6837	0.394	90259
May 2012	222	32152	48851	6841	0.399	88066
Jun 2012	174	29407	43573	6348	0.404	79502

Jul 2012	209	28869	42197	6078	0.408	77353
Aug 2012	220	28224	41234	5901	0.408	75579
Sep 2012	182	24994	37606	5475	0.401	68257
Oct 2012	182	25815	39294	6394	0.398	71685
Nov 2012	180	21343	34483	6841	0.384	62847
Dec 2012	142	16145	30175	10620	0.351	57082
Jan 2013	218	31079	50585	6657	0.382	88539
Feb 2013	134	27045	44180	5198	0.381	76557
Mar 2013	183	28786	49909	6377	0.367	85255
Apr 2013	204	28401	49558	6232	0.366	84395
May 2013	216	28847	46471	6318	0.385	81852
Jun 2013	218	26799	42839	5913	0.387	75769
Jul 2013	225	26665	41846	6069	0.391	74805
Aug 2013	206	26275	41443	6071	0.390	73995
Sep 2013	211	24216	38976	5920	0.385	69323
Oct 2013	228	23782	40359	6358	0.373	70727
Nov 2013	200	19400	36265	6953	0.351	62818
Dec 2013	177	15615	33029	11578	0.323	60399
Jan 2014	193	28851	53092	6992	0.354	89128
Feb 2014	189	24965	46226	5773	0.352	77153
Mar 2014	224	25861	51491	6897	0.336	84473
Apr 2014	232	24837	49441	7033	0.336	81543
May 2014	181	24970	47447	6932	0.346	79530
Jun 2014	146	23885	45367	6968	0.346	76366

Jul 2014	177	22976	44467	6987	0.342	74607
Aug 2014	172	22171	42270	6531	0.346	71144
Sep 2014	191	20109	40475	6312	0.334	67087
Oct 2014	210	20189	41380	6677	0.330	68456
Nov 2014	211	16611	36160	6967	0.318	59949
Dec 2014	197	14676	34341	10731	0.302	59945
Jan 2015	240	25234	52912	7100	0.325	85486
Feb 2015	163	20133	42711	5096	0.322	68103
Mar 2015	268	23608	53752	6776	0.308	84404
Apr 2015	243	22381	50436	6562	0.310	79622
May 2015	266	22426	48517	6700	0.319	77909
Jun 2015	203	22032	46558	6570	0.323	75363
Jul 2015	253	21545	45164	6573	0.326	73535
Aug 2015	210	20566	43211	6215	0.325	70202
Sep 2015	252	18789	40529	6016	0.320	65586
Oct 2015	243	18520	40280	6470	0.318	65513
Nov 2015	259	16031	37816	6582	0.301	60688
Dec 2015	225	13078	34028	10273	0.281	57604

References

1. Zhang LX. Pulmonary tuberculosis. In: Ye RG, Lu ZY, editors. Internal medicine. Beijing: People's Medical Publishing House Press; 2004. p.39-56.

2. National Bureau of Statistics of the People's Republic of China. Census data. http://www.stats.gov.cn/tjsj/pcsj/. Accessed 21 Apr 2018.
